# Supplementary material for: Endophytic Bacteria Colonizing the Petiole of the Desert Plant Zygophyllum dumosum Boiss: Possible Role in Mitigating Stress
Source: Plants (Basel). 2022 Feb 11;11(4):484. doi: 10.3390/plants11040484 (PMC8924888; doi:10.3390/plants11040484)
Supplement: Supplementary file 1 [file plants-11-00484-s001.zip › Figure .pdf]

Endophytic bacteria colonizing the petiole of the desert plant *Zygophyllum dumosum* Boiss: possible role in mitigating stress

Jansirani Srinivasan[1,2], Janardan Khadka[1], Nurit Novoplansky[1], Osnat Gillor[2] and Gideon Grafi[1*]

[1]French Associates Institute for Agriculture and Biotechnology of Drylands, [2]Zuckerberg Institute for Water Research, Jacob Blaustein Institutes for Desert Research, Ben-Gurion University of the Negev, Midreshet Ben Gurion 84990, Israel.

Figure S1. Calculating sodium concentration in winter and summer petioles

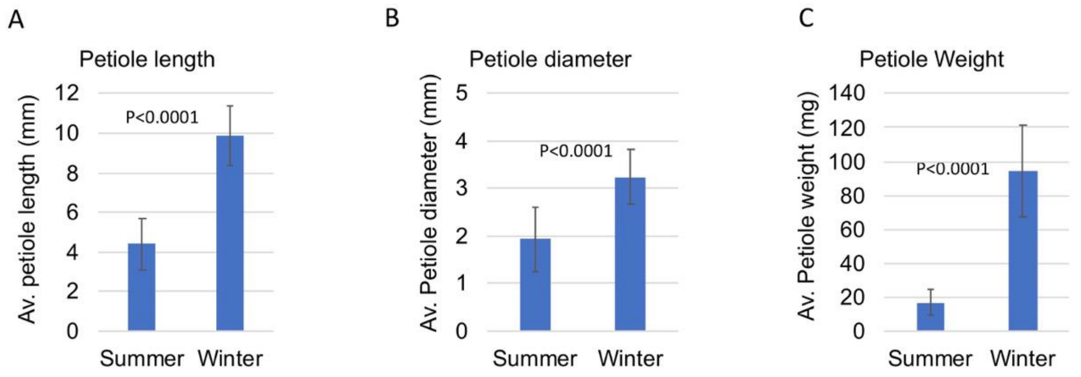

D

Petiole	Radius (r) (mm)	Length (L) (mm)	Volume (V) (cm^3) $V=\pi r^2 L$	Weight (pW) (mg)	# petioles In gFW	mg Na/ gFW	mg Na/ petiole	Na Conc. (M)
Summer	0.97	4.4	0.013	16.96	59	2.174	0.0368	0.123
Winter	1.62	9.88	0.081	94	10.6	1.561	0.147	0.079

$$\text{Na Conc. (mol/L)} = \frac{\text{Na (g)}}{\text{Volume (L)} \times \text{Molecular Weight (g/mol)}}$$

Fig. S1.petiole as a cylinder and determined its average volume (Vp=p r2 h) based on the measurements of petiole length (A), radius (B) and calculated sodium concentration based on petiole average weight (C). Statistical differences between treatments was perform by unpaired t test (GraphPad) and the p values are shown. (D) The data and formulas for calculation of Na concentration. Summer and winter petioles refer to petioles collected in October and April of 2010, respectively.

Figure S2. 16S rDNA sequencing

The list of 16S rDNA sequences of isolated endophytes. 16S rDNAs were PCR amplified using 1492-R and 27-F primers and each amplicon was sequenced in forward and reverse using the abovementioned primers. The F and R sequences were aligned and the combined seq data was used for NCBI blast analysis. Note, the sequence of one clone for each identified endophyte is shown.

> *J3 / Domibacillus robiginosus*

CTATACATGCAAGTCGAGCGGATTTAATTAGAGCTTGCTCTAATTAAATTACCGGCGGACGGGTGAGTAA
CACGTGGGCAACCTGCCCTGCAAATTGGGATAACTCCGGGAAACCGGGGCTAATACCGAATAACATTGG
AAACCTCATGGTTTCCTTTTAAAAGGCGGCTTCGGCTGTCACTGCAGGATGGGCCCGCGGCGCATTACCT
AGTTGGTGAGGTAACGGCTCACCAAGGCAACAATGCGTACCCAACCTGAAAGGGTGATCGGCCACACTG
GGACTGAAACACGGCCCAAACTCCTACGGGAGGCAGCAGTAGGGAATCTTCCGCAATGGACGAAAGTCT
GACGGAGCAACGCCCCGTGAGTGAAAAAGGTTTTCGGATCGTAAAACTCTGTTGTCAGGGAAGAACAAG
TACGGGAGTAACTGACCGTACCTTGACGGTACCTGGCCAGAAAGCCACGGCTAACTACTTGCCAGCACCC
GCGGTAATACGTAGGTGGCAAGCGTTGTCCGGAATTATTGGGCGTAAAGCGCGCGCAGGCGGCCTTTTA
AGTCTGATGTGAAAGCCCCCGGCTCAACCGGGGAGGGTCATTGGAAACTGGAAGGCTTGAGTGCAGAA
GAGAAGAGCGGAATTCCACGTGTAGCGGTGAAATGCGTAGAGATGTGGAGGAACACCAGTGGCGAAG
GCGGCTCTTTGGTCTGTAACTGACGCTGAGGCGCGAAAGCGTGGGGAGCGAACAGGATTAGATACCCTG
GTAGTCCACGCCGTAAACGATGAGTGCTAAGTGTTGGGGGGTTTCCGCCCCTCAGTGCTGCAGCTAACG
CATTAAGCACTCCGCCTGGGGAGTACGGCCGCAAGGCTGAAACTCAAAGGAATTGACGGGGGCCCGCA
CAAGCGGTGGAGCATGTGGTTTAATTCGAAGCAACGCGAAGAACCTTACCAGGTCTTGACATCCCGCTG
ACCGGTCTGGAGACAGATCTTCCCCTTCGGGGGCAGCGGTGACAGGTGGTGCATGGTTGTCGTCAGCTC
GTGTCGTGAGATGTTGGGTTAAGTCCCGCAACGAGCGCAACCCTTGATCTTAGTTGCCAGCATTCAGTTG
GGCACTCTAAGGTGACTGCCGGTGACAAACCGGAGGAAGGTGGGGATGACGTCAAATCATCATGCCCCT
TATGACCTGGGCTACACACGTGCTACAATGGATGGTACAAAGGGCTGCAAGACCGCGAGGTTTAGCCAA
TCCCATAAAACCATTCTCAGTTCGGATTGCAGGCTGCAACTCGCCTGCATGAAGCTGGAATCGCTAGTAA
TCGCGGATCAGCATGCCGCGGTGAATACGTTCCCGGGCCTTGTACACACCGCCCGTCACACCACGAGAGT
TTGCAACACCCGAAGTCGGTGGGGTAACCCTTACGGGAGCCAGCCGCCGAAG

> J6 / *Actinotalea ferrariae*

GCAGTCGAACGGTGATCTCGGTGCTTGCACCGGGTGATCAGTGGCGAACGGGTGAGTAACACGTGAGT
AACCTGCCCCCGACTCTGGGATAACCCCGGGAAATCGGAGCTAATACCGGATACGAGACGCACAGGCAT
CTGTAGCGTCTGGAAAGAATTTCGGTCGGGGATGGACTCGCGGCCTATCAGCTAGTTGGTGGGGTAACG
GCCCACCAAGGCGACGACGGGTAGCCGGCCTGAGAGGGCGACCGGCCACACTGGGACTGAGACACGG
CCCAGACTCCTACGGGAGGCAGCAGTGGGGAATATTGCACAATGGGCGAAAGCCTGATGCAGCGACGC
CGCGTGGGGGATGAAGGCCTTCGGGTTGTAAACCCCTTTCAGCAGGGAAGAAGCGAAAGTGACGGTAC
CTGCAGAAGAAGCGCCGGCTAACTACGTGCCAGCAGCCGCGGTAATACGTAGGGCGCAAGCGTTGTCC
GGAATTATTGGGCGTAAAGAGCTCGTAGGCGGTTTGTCGCGTCTGCTGTGAAAACCCGAGGCTCAACCT
CGGGCCTGCAGTGGGTACGGGCAGACTAGAGTGCGGTAGGGGAGACTGGAATTCCTGGTGTAGCGGTG
GAATGCGCAGATATCAGGAGGAACACCGATGGCGAAGGCAGGTCTCTGGGCCGCAACTGACGCTGAGG
AGCGAAAGCATGGGGAGCGAACAGGATTAGATACCCTGGTAGTCCATGCCGTAAACGTTGGGCACTAG
GTGTGGGGCTCATTCCACGAGTTCCGTGCCGCAGCAAACGCATTAAGTGCCCCGCCTGGGGAGTACGGC
CGCAAGGCTAAAACTCAAAGGAATTGACGGGGGCCCGCACAAGCGGCGGAGCATGCGGATTAATTCGA
TGCAACGCGAAGAACCTTACCAAGGCTTGACATATACCGAAAACTCATGGAGACATGGGGTCCGCAAGG
GCGGTATACAGGTGGTGCATGGTTGTCGTCAGCTCGTGTCGTGAGATGTTGGGTTAAGTCCCGCAACGA
GCGCAACCCTCGTCCTATGTTGCCAGCACATTATGGTGGGGACTCATAGGAGACTGCCGGGGTCAACTC
GGAGGAAGGTGGGGATGACGTCAAATCATCATGCCCCTTATGTCTTGGGCTTCACGCATGCTACAATGG
CCGGTACAAAGGGCTGCGATACCGCGAGGTGGAGCGAATCCCAAAAAGCCGGTCTCAGTTCGGATTGG
GGTCTGCAACTCGACCCCATGAAGTCGGAGTCGCTAGTAATCGCAGATCAGCAACGCTGCGGTGAATAC
GTTCCCGGGCCTTGTACACACCGCCCGTCAAGTCACGAAAGTCGGTAACACCCGAAGCCGGTGGCCCAA
CCCTTGGGAGGGAGCCGTCG

> J14 / *Bacillus jeotgali*

CTATACATGCAGTCGAGCGGATCTTCATTAGCTTGCTTTTGAAGATCAGCGGCGGACGGGTGAGTAACAC
GTGGGCAACCTGCCTGTAAGACTGGGATAACTTCGGGAAACCGGAGCTAATACCGGATAATCCTTTCCCT
CACATGAGGGAAAGCTGAAAGACGGTTTCGGCTGTCACTTACAGATGGGCCCGCGGCGCATTAGCTAGT
TGGTGAGGTAATGGCTCACCAAGGCAACGATGCGTAGCCGACCTGAGAGGGTGATCGGCCACACTGGG
ACTGAGACACGGCCCAGACTCCTACGGGAGGCAGCAGTAGGGAATCTTCCGCAATGGACGAAAGTCTG
ACGGAGCAACGCCGCGTGAACGATGAAGGCTTTCGGGTCGTAAAGTTCTGTTGTCAGGGAAGAACAAGT
ACCGGAGTAACTGCCGGTACCTTGACGGTACCTGACCAGAAAGCCACGGCTAACTACGTGCCAGCAGCC
GCGGTAATACGTAGGTGGCAAGCGTTGTCCGGAATTATTGGGCGTAAAGCGCGCGCAGGCGGTTCCTTA
AGTCTGATGTGAAAGCCCCCGGCTCAACCGGGGAGGGTCATTGGAAACTGGGGAACTTGAGTGCAGAA
GAGGAGAGCGGAATTCCACGTGTAGCGGTGAAATGCGTAGAGATGTGGAGGAACACCAGTGGCGAAG
GCGGCTCTCTGGTCTGTAACTGACGCTGAGGCGCGAAAGCGTGGGGAGCGAACAGGATTAGATACCCT
GGTAGTCCACGCCGTAAACGATGAGTGCTAAGTGTTAGAGGGTTTCCGCCCTTTAGTGCTGCAGCAAAC
GCATTAAGCACTCCGCCTGGGGAGTACGGCCGCAAGGCTGAAACTCAAAGGAATTGACGGGGGCCCGC
ACAAGCGGTGGAGCATGTGGTTTAATTCGAAGCAACGCGAAGAACCTTACCAGGTCTTGACATCCTCTGA
CAACCCTAGAGATAGGGCGTTCCCCTTCGGGGGACAGAGTGACAGGTGGTGCATGGTTGTCGTCAGCTC
GTGTCGTGAGATGTTGGGTTAAGTCCCGCAACGAGCGCAACCCTTGATCTTAGTTGCCAGCATTCAGTTG
GGCACTCTAAGGTGACTGCCGGTGACAAACCGGAGGAAGGTGGGGATGACGTCAAATCATCATGCCCCT
TATGACCTGGGCTACACACGTGCTACAATGGATGGAACAAAGGGTCGCGAAGCCGCGAGGTCGAGCCA
ATCCCATAAATCCATTCTCAGTTCGGATTGCAGGCTGCAACTCGCCTGCATGAAGCCGGAATCGCTAGTA
ATCGCGGATCAGCATGCCGCGGTGAATACGTTCCCGGGCCTTGTACACACCGCCCGTCACACCACGAGA
GTTTGTAACACCCGAAGTCGGTGGGGTAACCTTTTGGTAGCCAGCCGCCTAA

>J18 / *Bacillus licheniformis*

TGCAAGTCGAGCGGACCGACGGGAGCTTGCTCCCTTAGGTCAGCGGCGGACGGGTGAGTAACACGTGG
GTAACCTGCCTGTAAGACTGGGATAACTCCGGGAAACCGGGGCTAATACCGGATGCTTGATTGAACCGC
ATGGTTCAATCATAAAAGGTGGCTTTTAGCTACCACTTGCAGATGGACCCGCGGCGCATTAGCTAGTTGG
TGAGGTAACGGCTCACCAAGGCGACGATGCGTAGCCGACCTGAGAGGGTGATCGGCCACACTGGGACT
GAGACACGGCCCAGACTCCTACGGGAGGCAGCAGTAGGGAATCTTCCGCAATGGACGAAAGTCTGACG
GAGCAACGCCGCGTGAGTGATGAAGGTTTTCGGATCGTAAAACTCTGTTGTTAGGGAAGAACAAGTACC
GTTCGAATAGGGCGGCACCTTGACGGTACCTAACCAGAAAGCCACGGCTAACTACGTGCCAGCAGCCGC
GGTAATACGTAGGTGGCAAGCGTTGTCCGGAATTATTGGGCGTAAAGCGCGCGCAGGCGGTTTCTTAAG
TCTGATGTGAAAGCCCCCGGCTCAACCGGGGAGGGTCATTGGAAACTGGGGAACTTGAGTGCAGAAGA
GGAGAGTGGAATTCCACGTGTAGCGGTGAAATGCGTAGAGATGTGGAGGAACACCAGTGGCGAAGGC
GACTCTCTGGTCTGTAACTGACGCTGAGGCGCGAAAGCGTGGGGAGCGAACAGGATTAGATACCCTGGT
AGTCCACGCCGTAAACGATGAGTGCTAAGTGTTAGAGGGTTTCCGCCCTTTAGTGCTGCAGCAAACGCAT
TAAGCACTCCGCCTGGGGAGTACGGTCGCAAGACTGAAACTCAAAGGAATTGACGGGGGCCCGCACAA
GCGGTGGAGCATGTGGTTTAATTCGAAGCAACGCGAANAACCTTACCNNTCTTGACATCCTCTGACNAC
CCTAGAGATAGGGCTTCCCCTTCGGGGGCAGAGTGACNGNTGGNGCATGGNNTCGTCAGCTCNNGTC

>J21 / *Brevibacillus halotolerans*

TGCAGTCGAGCGAGGGTTTTCGGACCCTAGCGGCGGACGGGTGAGTAACACGTAGGCAACCTGCCTGTA
AGACTGGGATAACATAGGGAAACTTATGCTAATACCGGATAGGGTTTTGCTTCGCATGAAGCGAAACGG
AAAGATGGCGCAAGCTATCACTTGCAGATGGGCCTGCGGCGCATTAGCTAGTTGGTGAGGTAAAGGCTC
ACCAAGGCGACGATGCGTAGCCGACCTGAGAGGGTGACCGGCCACACTGGGACTGAGACACGGCCCAG
ACTCCTACGGGAGGCAGCAGTAGGGAATTTTCCACAATGGACGAAAGTCTGATGGAGCAACGCCGCGTG
AACGATGAAGGCTTTCGGGTCGTAAAGTTCTGTTGTTAGGGAAGAAACAGTGCCATTTGAATAAGATGG
CGCCTTGACGGTACCTAACGAGAAAGCCACGGCTAACTACGTGCCAGCAGCCGCGGTAATACGTAGGTG
GCAAGCGTTGTCCGGAATTATTGGGCGTAAAGCGCGCAGGTGGCTATGTAAGTCTGATGTTAAAGCC
CGAGGCTCAACCTCGGTTCGCATTGGAAACTGCGTAGCTTGAGTGCAGGAGAGGAAAGTGGTATTCCAC
GTGTAGGTATTCCACGTGTAGCGGTGAAATGCGTAGAGATGTGGAGGAACACCAGTGGCGAAGGCGAC
TTTCTGGCCTGTAACTGACACTGAGGCGCGAAAGCGTGGGGAGCAAACAGGATTAGATACCCTGGTAGT
CCACGCCGTAAACGATGAGTGCTAGGTGTTAGGGGTTTCAATACCCTTAGTGCCGCAGCTAACGCAATAA
GCACTCCGCCTGGGGAGTACGCTCGCAAGAGTGAAACTCAAAGGAATTGACGGGGGCCCGCACAAGCG
GTGGAGCATGTGGTTTAATTCGAAGCAACGCGAAGAACCTTACCAGGTCTTGACATCCCACTGACCGCTC
TAGAGATAGAGCTTCCCTTCGGGGCAGTGGTGACAGGTGGTGCATGGTTGTCGTCAGCTCGTGTCGTGA
GATGTTGGGTTAAGTCCCGCAACGAGCGCAACCCTTATCTTTAGTTGCCAGCATTCAGTTGGGCACTCTA
GAGAGACTGCCGTCGACAAGACGGAGGAAGGCGGGGATGACGTCAAATCATCATGCCCCTTATGACCT
GGGCTACACACGTGCTACAATGGTTGGTACAACGGGATGCTACTTCGCGAGAAGATGCTAATCTCTTAAA
ACCAATCTCAGTTCGGATTGTAGGCTGCAACTCGCCTACATGAAGTCGGAATCGCTAGTAATCGCGGATC
AGCATGCCGCGGTGAATACGTTCCCGGGCCTTGTACACACCGCCCGTCACACCACGGGAGTTTGCAACAC
CCGAAGTCGGTGAGGTAACCGCAAGGAGCCAGCCGC

>J23 / *Brevibacterium frigoritolerans*

TGCAGTCGAGCGAATCGATGGGAGCTTGCTCCCTGAGATTAGCGGCGGACGGGTGAGTAACACGTGGG
CAACCTGCCTATAAGACTGGGATAACTTCGGGAAACCGGAGCTAATACCGGATACGTTCTTTTCTCGCAT
GAGAGAAGATGGAAAGACGGTTTACGCTGTCACTTATAGATGGGCCCGCGGCGCATTAGCTAGTTGGTG
AGGTAATGGCTCACCAAGGCGACGATGCGTAGCCGACCTGAGAGGGTGATCGGCCACACTGGGACTGA
GACACGGCCCAGACTCCTACGGGAGGCAGCAGTAGGGAATCTTCCGCAATGGACGAAAGTCTGACGGA
GCAACGCCGCGTGAACGAAGAAGGCCTTCGGGTCGTAAAGTTCTGTTGTTAGGGAAGAACAAGTACCAG
AGTAACTGCTGGTACCTTGACGGTACCTAACCAGAAAGCCACGGCTAACTACGTGCCAGCAGCCGCGGT
AATACGTAGGTGGCAAGCGTTGTCCGGAATTATTGGGCGTAAAGCGCGCGCAGGTGGTTCCTTAAGTCT
GATGTGAAAGCCCACGGCTCAACCGTGGAGGGTCATTGGAAACTGGGGAACTTGAGTGCAGAAGAGGA
AAGTGGAATTCCAAGTGTAGCGGTGAAATGCGTAGAGATTTGGAGGAACACCAGTGGCGAAGGCGACT
TTCTGGTCTGTAACTGACACTGAGGCGCGAAAGCGTGGGGAGCAAACAGGATTAGATACCCTGGTAGTC
CACGCCGTAAACGATGAGTGCTAAGTGTTAGAGGGTTTCCGCCCTTTAGTGCTGCAGCTAACGCATTAAG
CACTCCGCCTGGGGAGTACGGCCGCAAGGCTGAAACTCAAAGGAATTGACGGGGGCCCGCACAAGCGG
TGGAGCATGTGGTTTAATTCGAAGCAACGCGAAGAACCTTACCAGGTCTTGACATCCTCTGACAACCCTA
GAGATAGGGCTTTCCCCTTCGGGGGACAGAGTGACAGGTGGTGCATGGTTGTCGTCAGCTCGTGTCGTG
AGATGTTGGGTTAAGTCCCGCAACGAGCGCAACCCTTGATCTTAGTTGCCAGCATTCAGTTGGGCACTCT
AAGGTGACTGCCGGTGACAAACCGGAGGAAGGTGGGGATGACGTCAAATCATCATGCCCCTTATGACCT
GGGCTACACACGTGCTACAATGGATGGTACAAAGGGCTGCAAACCTGCGAAGGTAAGCGAATCCCATAA
AGCCATTCTCAGTTCGGATTGCAGGCTGCAACTCGCCTGCATGAAGCCGGAATCGCTAGTAATCGCGGAT
CAGCATGCCGCGGTGAATACGTTCCCGGGCCTTGTACACACCGCCCGTCACACCACGAGAGTTTGTAACA
CCCGAAGTCGGTGAGGTAACCTTCATGGAGCCAGCCGCCTA

>J25 / *Fictibacillus nanhaiensis*

ATGCAAGTCGAGCGAATGACGAGGAGCTTGCTCCTCTGATTTAGCGGCGGACGGGTGAGTAACACGTG
GGTAATCTGCCTGTAAGACGGGGATAACTCCGGGAAACCGGGGCTAATACCGGATAACAAGAGAAGAT
GCATATCTTCTTTTTGAAAGTCGGTTTCGGCTGACACTTACAGATGAGCCCGCGGCGCATTAGCTAGTTG
GTGAGGTAACGGCTCACCAAGGCGACGATGCGTAGCCGACCTGAGAGGGTGATCGGCCACACTGGGAC
TGAGACACGGCCCAGACTCCTACGGGAGGCAGCAGTAGGGAATCTTCGGCAATGGGCGAAAGCCTGAC
CGAGCAACGCCGCGTGAGCGATGAAGGCCTTCGGGTCGTAAAGCTCTGTTGTTAGAGAAGAACAAGTAC
GAGAGTAACTGCTCGTACCTTGACGGTACCTAACCAGAAAGCCACGGCTAACTACGTGCCAGCAGCCGC
GGTAATACGTAGGTGGCAAGCGTTATCCGGAATTATTGGGCGTAAAGCGCGCGCAGGCGGTCTCTTAAG
TCTGATGTGAAAGCCCACGGCTCAACCGTGGAGGGTCATTGGAAACTGGGAGACTTGAGTGCAGGAGA
GAAAAGTGGAATTCCACGTGTAGCGGTGAAATGCGTAGAGATGTGGAGGAACACCAGTGGCGAAGGCG
GCTTTTTGGCCTGTAACTGACGCTGAGGCGCGAAAGCGTGGGGAGCAAACAGGATTAGATACCCTGGTA
GTCCACGCCGTAAACGATGAGTGCTAGGTGTTGGGGGGTTCCACCCTCAGTGCTGAAGTTAACACATTAA
GCACTCCGCCTGGGGAGTACGACCGCAAGGTTGAAACTCAAAGGAATTGACGGGGGCCCGCACAAGCA
GTGGAGCATGTGGTTTAATTCGAAGCAACGCGAAGAACCTTACCAGGTCTTGACATCCTCTGACCACTCT
AGAGATAGAGCTTTCCCCTTCGGGGGACAGAGTGACAGGTGGTGCATGGTTGTCGTCAGCTCGTGTCGT
GAGATGTTGGGTTAAGTCCCGCAACGAGCGCAACCCTTGACCTTAGTTGCCAGCATTCAGTTGGGCACTC
TAAGGTGACTGCCGGTGACAAACCGGAGGAAGGTGGGGATGACGTCAAATCATCATGCCCCTTATGACC
TGGGCTACACACGTGCTACAATGGATGGTACAAAGGGTTGCGAAGCCGCGAGGCCAAGCCAATCCCAAA
AAGCCATTCTCAGTTCGGATTGTAGGCTGCAACTCGCCTACATGAAGCCGGAATTGCTAGTAATCGCGGA
TCAGCATGCCGCGGTGAATACGTTCCCGGGCCTTGTACACACCGCCCGTCACACCACGAGAGTTTGTAAC
ACCCGAAGTCGGTGGGGTAACCCTCTGGGAGCCAGCCGCCGAAGGTG

>J27 / *Bacillus megaterium*

AATACATGCAAAGTCGAGCGAACTGATTAGAAGCTTGCTTCTATGACGTTAGCGGCGGACGGGTGAGTA
ACACGTGGGCAACCTGCCTGTAAGACTGGGATAACTTCGGGAAACCGAAGCTAATACCGGATAGGATCT
TCTCCTTCATGGGAGATGATTGAAAGATGGTTTCGGCTATCACTTACAGATGGGCCCGCGGTGCATTAGC
TAGTTGGTGAGGTAACGGCTCACCAAGGCAACGATGCATAGCCGACCTGAGAGGGTGATCGGCCACACT
GGGACTGAGACACGGCCCAGACTCCTACGGGAGGCAGCAGTAGGGAATCTTCCGCAATGGACGAAAGT
CTGACGGAGCAACGCCGCGTGAGTGATGAAGGCTTTCGGGTCGTAAAACTCTGTTGTTAGGGAAGAACA
AGTACGAGAGTAACTGCTTGTACCTTGACGGTACCTAACCAGAAAGCCACGGCTAACTACGTGCCAGCA
GCCGCGGTAATACGTAGGTGGCAAGCGTTATCCGGAATTATTGGGCGTAAAGCGCGCGCAGGCGGTTTC
TTAAGTCTGATGTGAAAGCCCACGGCTCAACCGTGGAGGGTCATTGGAAACTGGGGAACTTGAGTGCAG
AAGAGAAAAGCGGAATTCCACGTGTAGCGGTGAAATGCGTAGAGATGTGGAGGAACACCAGTGGCGAA
GGCGGCTTTTTGGTCTGTAACTGACGCTGAGGCGCGAAAGCGTGGGGAGCAAACAGGATTAGATACCCT
GGTAGTCCACGCCGTAAACGATGAGTGCTAAGTGTTAGAGGGTTTCCGCCCTTTAGTGCTGCAGCTAACG
CATTAAGCACTCCGCCTGGGGAGTACGGTCGCAAGACTGAAACTCAAAGGAATTGACGGGGGCCCGCAC
AAGCGGTGGAGCATGTGGTTTAATTCGAAGCAACGCGAAGAACCTTACCAGGTCTTGACATCCTCTGACA
ACTCTAGAGATAGAGCGTTCCCCTTCGGGGGACAGAGTGACAGGTGGTGCATGGTTGTCGTCAGCTCGT
GTCGTGAGATGTTGGGTTAAGTCCCGCAACGAGCGCAACCCTTGATCTTAGTTGCCAGCATTTAGTTGGG
CACTCTAAGGTGACTGCCGGTGACAAACCGGAGGAAGGTGGGGATGACGTCAAATCATCATGCCCCTTA
TGACCTGGGCTACACACGTGCTACAATGGATGGTACAAAGGGCTGCAAGACCGCGAGGTCAAGCCAATC
CCATAAAACCATTCTCAGTTCGGATTGTAGGCTGCAACTCGCCTACATGAAGCTGGAATCGCTAGTAATC
GCGGATCAGCATGCCGCGGTGAATACGTTCCCGGGCCTTGTACACACCGCCCGTCACACCACGAGAGTTT
GTAACACCCGAAGTCGGTGGAGTAACCGTAAGGAGCTAGCCGC

>J29 / *Pseudokineococcus basanitobsidens*

ATGCAGTCGAACGGTGATGCCCCGGCTTGCCGGGGTGGATCAGTGGCGAACGGGTGAGTAACACGTGA
GCAACCTGCCCCCGACTCTGGGATAACCACCGGAAACGGTGGCTAATACCGGATATGACGGCTGGCCGC
ATGGTCTGGTCGTGGAAAGTTTCTCGGTTGGGGATGGGCTCGCGGCCTATCAGCTTGTTGGTGGGGTGA
TGGCCTACCAAGGCGACGACGGGTAGCCGGCCTGAGAGGGCGACCGGCCACACTGGGACTGAGACACG
GCCCAGACTCCTACGGGAGGCAGCAGTGGGGAATATTGCGCAATGGGCGGAAGCCTGACGCAGCGACG
CCGCGTGAGGGATGAAGGCCTTCGGGTTGTAAACCTCTTTCAGCTCCGAAGAAGCGAGAGTGACGGTAG
GAGCAGAAGAAGCACCGGCTAACTACGTGCCACCAGCCGCGGTAATACGTAGGGTGCAAGCGTTGTCC
GGAATTATTGGGCGTAAAGAGCTCGTAGGCGGCTCGTCGCGTCTGCTGTGAAAGCCTAGGGCTTAACCC
TGGGTCTGCAGTGGGTACGGGCGAGCTAGAGTGCGGTAGGGGAGACTGGAATTCCTGGTGTAGCGGTG
AAATGCGCAGATATCAGGAGGAACACCGGTGGCGAAGGCGGGTCTCTGGGCCGTAACTGACGCTGAGG
AGCGAAAGCATGGGTAGCGAACAGGATTAGATACCCTGGTAGTCCATGCCGTAAACGTTGGGCGCTAG
GTGTGGGCCGCATTCCACGTGGTCCGTGCCGCAGCTAACGCATTAAGCGCCCCGCCTGGGGAGTACGGC
CGCAAGGCTAAACTCAAAGGAATTGACGGGGCCCCGCACAAGCGGCGGAGCATGCGGATTAATTCGAT
GCAACGCGAAGAACCTTACCAAGGCTTGACATGCAGGGAAATCCTCCAGAGATGGTGGGTCCGCAAGG

GTCCTGCGCAGGTGGTGCATGGTTGTCGTCAGCTCGTGTCGTGAGATGTTGGGTTAAGTCCCGCAACGA
GCGCAACCCTCGTTCGATGTTGCCAGCGGGTGATGCCGGGGACTCATCGGAGACTGCCGGGGTCAACTC
GGAGGAAGGTGGGGATGACGTCAAATCATCATGCCCCTTATGTCTTGGGCTTCACGCATGCTACAATGG
CCAGTACAATGGGCTGCGAGACCGTGAGGTGGAGCGAATCCCAAAAAGCTGGTCTCAGTTCGGATTGG
GGTCTGCAACTCGACCCCATGAAGTCGGAGTCGCTAGTAATCGCAGATCAGCAACGCTGCGGTGAATAC
GTTCCCGGGGCTTGTACACACCGCCCGTCACGTCACGAAAGTCGGTAACACCCGAAGCCGGTGGCCCAA
CCCCTTGTGGGAGGGAGC

>J33 / *Arthrobacter subterraneus*

CACATGCAGTCGAACGATGATCTCCAGCTTGCTGGGGGGATTAGTGGCGAACGGGTGAGTAACACGTGA
GTAACCTGCCCTTGACTCTGGGATAAGCCTGGGAAACTGGGTCTAATACTGGATACGACTCTTCACCGCA
TGGTGGGGGGTGGAAAGCTTTTGTGGTTTTGGATGGACTCGCGGCCTATCAGCTTGTTGGTGGGGTAAT
GGCCTACCAAGGCGACGACGGGTAGCCGGCCTGAGAGGGTGGACGGCCACACTGGGACTGAGACACG
GCCCAGACTCCTACGGGAGGCAGCAGTGGGGAATATTGCACAATGGGCGCAAGCCTGATGCAGCGACG
CCGCGTGAGGGATGAAGGCCTTCGGGTTGTAAACCTCTTTCAGTAGGGAAGAAGCGAAAGTGACGGTA
CCTGCAGAAGAAGCGCCGGCTAACTACGTGCCAGCAGCCGCGGTAATACGTAGGGCGCAAGCGTTATCC
GGAATTATTGGGCGTAAAGAGCTCGTAGGCGGTTTGTCGCGTCTGCCGTGAAAGTCCGGGGCTTAACTC
CGGATCTGCGGTGGGTACGGGCAGACTTGAGTGATGTAGGGGAGACTGGAATTCCTGGTGTAGCGGTG
AAATGCGCAGATATCAGGAGGAACACCGATGGCGAAGGCAGGTCTCTGGGCATTAACTGACGCTGAGG
AGCGAAAGCATGGGGAGCGAACAGGATTAGATACCCTGGTAGTCCATGCCGTAAACGTTGGGCACTAG
GTGTGGGGGACATTCCACGTTTTCCGCGCCGTAGCTAACGCATTAAGTGCCCCGCCTGGGGAGTACGGC
CGCAAGGCTAAAACTCAAAGGAATTGACGGGGGCCCGCACAAGCGGCGGAGCATGCGGATTAATTCGA
TGCAACGCGAAGAACCTTACCAAGGCTTGACATGAACCGGAACGGCGCAGAGATGTGTCGGCCACTTGT
GGCCGGTTTACAGGTGGTGCATGGTTGTCGTCAGCTCGTGTCGTGAGATGTTGGGTTAAGTCCCGCAAC
GAGCGCAACCCTCGTTCCATGTTGCCAGCGGGTTATGCCGGGGACTCATGGGAGACTGCCGGGGTCAAC
TCGGAGGAAGGTGGGGACGACGTCAAATCATCATGCCCCTTATGTCTTGGGCTTCACGCATGCTACAATG
GCCGGTACAAAGGGTTGCGATGCTGTGAGGTGGAGCTAATCCCAAAAAGCCGGTCTCAGTTCGGATTGA
GGTCTGCAACTCGACCTCATGAAGTCGGAGTCGCTAGTAATCGCAGATCAGCAACGCTGCGGTGAATAC
GTTCCCGGGCCTTGTACACACCGCCCGTCAAGTCACGAAAGTTGGTAACACCCGAAGCCGGTGGCCTAAC
CCCTTGTGGGAGGGAGCCGTCGA

>J36 / *Kocuria rosea*

TGCAGTCGAACGATGATGCCCAGCTTGCTGGGCGGATTAGTGGCGAACGGGTGAGTAATACGTGAGTA
ACCTGCCCTTGACTCTGGGATAAGCCTGGGAAACTGGGTCTAATACTGGATACTACTTCCTGCCGCATGG
TGGGTGGTGGAAAGGGTTTTACTGGTTTTGGATGGGCTCACGGCCTATCAGCTTGTTGGTGGGGTAATG
GCTCACCAAGGCGACGACGGGTAGCCGGCCTGAGAGGGTGACCGGCCACACTGGGACTGAGACACGGC
CCAGACTCCTACGGGAGGCAGCAGTGGGGAATATTGCACAATGGGCGGAAGCCTGATGCAGCGACGCC
GCGTGAGGGATGACGGCCTTCGGGTTGTAAACCTCTTTCAGTAGGGAAGAAGCGAGAGTGACGGTACCT
GCAGAAGAAGCGCCGGCTAACTACGTGCCAGCAGCCGCGGTAATACGTAGGGCGCAAGCGTTGTCCGG
AATTATTGGGCGTAAAGAGCTCGTAGGCGGTTTGTCGCGTCTGCTGTGAAAGCCCGGGGCTCAACCCCG
GGTCTGCAGTGGGTACGGGCAGACTAGAGTGCAGTAGGGGAGACTGGAATTCCTGGTGTAGCGGTGAA
ATGCGCAGATATCAGGAGGAACACCGATGGCGAAGGCAGGTCTCTGGGCTGTTACTGACGCTGAGGAG
CGAAAGCATGGGGAGCGAACAGGATTAGATACCCTGGTAGTCCATGCCGTAAACGTTGGGCACTAGGT
GTGGGGGACATTCCACGTTTTCCGCGCCGTAGCTAACGCATTAAGTGCCCCGCCTGGGGAGTACGGCCG
CAAGGCTAAAACTCAAAGGAATTGACGGGGGCCCGCACAAGCGGCGGAGCATGCGGATTAATTCGATG
CAACGCGAAGAACCTTACCAAAGAACCTTACCAAGGCTTGACATTCACCGGACCGCCCCAGAGATGGGG
TTTCCCTTCGGGGCTGGTGGACAGGTGGTGCATGGTTGTCGTCAGCTCGTGTCGTGAGATGTTGGGTTAA
GTCCCGCAACGAGCGCAACCCTCGTTCTATGTTGCCAGCACGTGATGGTGGGGACTCATAGGAGACTGC
CGGGGTCAACTCGGAGGAAGGTGGGGATGACGTCAAATCATCATGCCCCTTATGTCTTGGGCTTCACGC
ATGCTACAATGGCCGGTACAAAGGGTTGCGATACTGTGAGGTGGAGCTAATCCCAAAAAGCCGGTCTCA
GTTCGGATTGAGGTCTGCAACTCGACCTCATGAAGTCGGAGTCGCTAGTAATCGCAGATCAGCAACGCT
GCGGTGAATACGTTCCCGGGCCTTGTACACACCGCCCGTCAAGTCACGAAAGTTGGTAACACCCGAAGC
CGGTGGCCTA

>J37 / *Bacillus thioparans*

ACATGCAAGTCGAGCGGATCTTCATTAGCTTGCTTTTGAAGATCAGCGGCGGACGGGTGAGTAACACGT
GGGCAACCTGCCTGTAAGACTGGGATAACTTCGGGAAACCGGAGCTAATACCGGATAATCCTTTCCCTCA
CATGAGGGAAAGCTGAAAGACGGTTTCGGCTGTCACTTACAGATGGGCCCGCGGCGCATTAGCTAGTTG
GTGAGGTAACGGCTCACCAAGGCAACGATGCGTAGCCGACCTGAGAGGGTGATCGGCCACACTGGGAC
TGAGACACGGCCCAGACTCCTACGGGAGGCAGCAGTAGGGAATCTTCCGCAATGGACGAAAGTCTGAC
GGAGCAACGCCGCGTGAACGATGAAGGCTTTCGGGTCGTAAAGTTCTGTTGTCAGGGAAGAACAAGTAC
CGGAGTAACTGCCGGTACCTTGACGGTACCTGACCAGAAAGCCACGGCTAACTACGTGCCAGCAGCCGC
GGTAATACGTAGGTGGCAAGCGTTGTCCGGAATTATTGGGCGTAAAGCGCGCGCAGGCGGTTCCTTAAG
TCTGATGTGAAAGCCCCCGGCTCAACCGGGGAGGGTCATTGGAAACTGGGGAACTTGAGTGCAGAAGA
GGAGAGCGGAATTCCACGTGTAGCGGTGAAATGCGTAGAGATGTGGAGGAACACCAGTGGCGAAGGC
GGCTCTCTGGTCTGTAACTGACGCTGAGGCGCGAAAGCGTGGGGAGCGAACAGGATTAGATACCCTGGT
AGTCCACGCCGTAAACGATGAGTGCTAAGTGTTAGAGGGTTTCCGCCCTTTAGTGCTGCAGCAAACGCAT
TAAGCACTCCGCCTGGGGAGTACGGCCGCAAGGCTGAAACTCAAAGGAATTGACGGGGGCCCGCACAA
GCGGTGGAGCATGTGGTTTAATTCGAAGCAACGCGAAGAACCTTACCAGGTCTTGACATCCTCTGACAAC
CCTAGAGATAGGGCGTTCCCCTTCGGGGGACAGAGTGACAGGTGGTGCATGGTTGTCGTCAGCTCGTGT
CGTGAGATGTTGGGTTAAGTCCCGCAACGAGCGCAACCCTTGATCTTAGTTGCCAGCATTCAGTTGGGCA
CTCTAAGGTGACTGCCGGTGACAAACCGGAGGAAGGTGGGGATGACGTCAAATCATCATGCCCCTTATG
ACCTGGGCTACACACGTGCTACAATGGATGGAACAAAGGGTCGCGAAGCCGCGAGGTGAAGCCAATCC
CATAAATCCATTCTCAGTTCGGATTGCAGGCTGCAACTCGCCTGCATGAAGCCGGAATCGCTAGTAATCG
CGGATCAGCATGCCGCGGTGAATACGTTCCCGGGCCTTGTACACACCGCCCGTCACACCACGAGAGTTTG
TAACACCCGAAGTCGGTGGGGTAACCTTTTGGAGCCAGCCGC

>J40 / *Bacillus sinesaloumensis*

ATACATGCAAGTCGAGCGGATCTTTGGGAGCTTGCTCCCAAAGGTTAGCGGCGGACGGGTGAGTAACAC
GTGGGTAACCTGCCTGTAAGACTGGGATAACTTCGGGAAACCGGAGCTAATACCGGATAACATATAAGA
CCACATGGTCTTATATTAAAAGGTGGCTTTTAGCTACCACTTACAGATGGACCCGCGGCGCATTAGCTAG
TTGGTGAGGTAACGGCTCACCAAGGCGACGATGCGTAGCCGACCTGAGAGGGTGATCGGCCACACTGG
GACTGAGACACGGCCCAGACTCCTACGGGAGGCAGCAGTAGGGAATCTTCCGCAATGGACGAAAGTCT
GACGGAGCAACGCCGCGTGAGCGATGAAGGCCTTCGGGTCGTAAAGCTCTGTTGTTAGGGAAGAACAA
GTATCGTTCGAATAGGGCGGTACCTTGACGGTACCTAACCAGAAAGCCACGGCTAACTACGTGCCAGCA
GCCGCGGTAATACGTAGGTGGCAAGCGTTATCCGGAATTATTGGGCGTAAAGCGCGCGCAGGCGGTCCT
TTAAGTCTGATGTGAAAGCCCACGGCTCAACCGTGGAGGGTCATTGGAAACTGGGGGACTTGAGTGCAG
AAGAGGAGAGCGGAATTCCACGTGTAGCGGTGAAATGCGTAGAGATGTGGAGGAACACCAGTGGCGA
AGGCGGCTCTCTGGTCTGTAACTGACGCTGAGGCGCGAAAGCGTGGGGAGCGAACAGGATTAGATACC
CTGGTAGTCCACGCCGTAAACGATGAGTGCTAAGTGTTAGAGGGTTTCCGCCCTTTAGTGCTGCAGCAAA
CGCATTAAGCACTCCGCCTGGGGAGTACGGTCGCAAGACTGAAACTCAAAGGAATTGACGGGGGCCCG
CACAAGCGGTGGAGCATGTGGTTTAATTCGAAGCAACGCGAAGAACCTTCCCATGTCTTGACCTCCCATG
ACAACTCTAGAGATAGGGTTTTCCCTTGGGGGACATGGTGACAGGTGGTGCATGGTTGTCGTCAGCTCG
TGTCGTGAGATGTTGGGTTAAGTCCCGCAACGAGCGCAACCCTTGATCTTAGTTGCCAGCATTCAGTTGG
GCACTCTAAGGTGACTGCCGGTGACAAACCGGAGGAAGGTGGGGATGACGTCAAATCATCATGCCCCTT
ATGACCTGGGCTACACACGTGCTACAATGGATGGTACAAAGGGCAGCGAAACCGCGAGGTTAAGCAAA
TCCCATAAAACCATTCTCAGTTCGGATTGCAGGCTGCAACTCGCCTGCATGAAGCCGGAATCGCTAGTAA
TCGCGGATCAGCATGCCGCGGTGAATACGTTCCCGGGCCTTGTACACACCGCCCGTCACACCACGAGAGT
TTGTAACACCCGAAGTCGGTGGGGTAACCGTAAGGAGCCAGCCGC

>J42 / *Arthrobacter agilis*

GCAGAAGAAGCGCCNGGCTAACTACGTGCCAGCAGCCGCGGTAATACGTAGGGCGCAAGCGTTATCCG
GGAATTATTGGGCGTAAAGAGCTCGTAGGCGGTTTGTCGCGTCTGCCGTGAAAGTCCGGGGCTTAACCC
CGGATCTGCGGTGGGTACGGGCAGACTAGAGTGCAGTAGGGGAGACTGGAATTCCTGGTGTAGCGGTG
AAATGCGCAGATATCAGGAGGAACACCGATGGCGAAGGCAGGTCTCTGGGCTGTAACTGACGCTGAGG
AGCGAAAGCATGGGGAGCGAACAGGATTAGATACCCTGGTAGTCCATGCCGTAAACGTTGGGCACTAG
GTGTGGGGGACATTCCACGTTTTCCGCGCCGTAGCTAACGCATTAAGTGCCCCGCCTGGGGAGTACGGC
CGCAAGGCTAAAACTCAAAGGAATTGACGGGGGCCCGCACAAGCGGCGGAGCATGCGGATTAATTCGA
TGCAACGCGAAGAACCTTACCAAGGCTTGACATGAACCGGAATGATGCAGAGATGTGTCAGCCACTTGT
GGCCGGTTTACAGGTGGTGCATGGTTGTCGTCAGCTCGTGTCGTGAGATGTTGGGTTAAGTCCCGCAAC
GAGCGCAACCCTCGTTCCATGTTGCCAGCGGGTTATGCCGGGGACTCATGGGAGACTGCCGGGGTCAAC
TCGGAGGAAGGTGGGGACGACGTCAAATCATCATGCCCCTTATGTCTTGGGCTTCACGCATGCTACAATG
GCCGGTACAAAGGGTTGCGATACTGTGAGGTGGAGCTAATCCCAAAAAGCCGGTCTCAGTTCGGATTGA
GGTCTGCAACTCGACCTCATGAAGTTGGAGTCGCTAGTAATCGCAGATCAGCAACGCTGCGGTGAATAC
GTTCCCGGGCCTTGTACACACCGCCCGTCAAGTCACGAAAGTTGGTAACACCCGAAGCCGGTGGCCTAAC
CCCTTGTGGGAGGGAGCCGTCG

>J43 / *Dietzia lutea*

CATGCAGTCGAACGGTAAGGCCCTTACAGGGGTACACGAGTGGCGAACGGGTGAGTAACACGTGGGTA
ATCTGCCCTGCACTTCGGGATAAGCCTGGGAAACTGGGTCTAATACCGGATATTCAACTTTCGCCGCATG
GTGGTGGTTGGAAAGTTTTTCGGTGCAGGATGAGCCCGCGGCCTATCAGCTTGTTGGTGGGGTAATGGC
CTACCAAGGCGACGACGGGTAGCCGGCCTGAGAGGGTGATCGGCCACACTGGGACTGAGACACGGCCC
AGACTCCTACGGGAGGCAGCAGTGGGGAATATTGCACAATGGGCGGAAGCCTGATGCAGCGACGCCGC
GTGGGGGATGACGGTCTTCGGATTGTAAACCCCTTTCAGTAGGGACGAAGCGCAAGTGACGGTACCTGC
AGAAGAAGCACCGGCCAACTACGTGCCAGCAGCCGCGGTAATACGTAGGGTGCGAGCGTTGTCCGGAA
TTACTGGGCGTAAAGAGCTCGTAGGCGGTTTGTCACGTCGTCTGTGAAATCCTCCAGCTTAACTGGGGGC
GTGCAGGCGATACGGGCAGACTTGAGTACTACAGGGGAGACTGGAATTCCTGGTGTAGCGGTGAAATG
CGCAGATATCAGGAGGAACACCGGTGGCGAAGGCGGGTCTCTGGGTAGTAACTGACGCTGAGGAGCGA
AAGCATGGGTAGCGAACAGGATTAGATACCCTGGTAGTCCATGCCGTAAACGGTGGGCGCTAGGTGTG
GGGTCCTTCCACGGATTCCGTGCCGTAGCTAACGCATTAAGCGCCCCGCCTGGGGAGTACGGCCGCAAG
GCTAAAACTCAAAGGAATTGACGGGGGCCCGCACAAGCGGCGGAGCATGTGGATTAATTCGATGCAAC
GCGAAGAACCTTACCTAGGCTTGACATATACAGGACGACGGCAGAGATGTCGTTTCCCTTGTGGCTTGTA
TACAGGTGGTGCATGGTTGTCGTCAGCTCGTGTCGTGAGATGTTGGGTTAAGTCCCGCAACGAGCGCAA
CCCCTGTCTCATGTTGCCAGCACGTTATGGTGGGGACTCGTGAGAGACTGCCGGGGTCAACTCGGAGGA
AGGTGGGGATGACGTCAAATCATCATGCCCCTTATGTCTAGGGCTTCACACATGCTACAATGGCTAGTAC
AGAGGGCTGCGATACCGTGAGGTGGAGCGAATCCCTTAAAGCTGGTCTCAGTTCGGATTGGGGTCTGCA
ACTCGACCCCATGAAGTCGGAGTCGCTAGTAATCGCAGATCAGCAACGCTGCGGTGAATACGTTCCCGG
GCCTTGTACACACCGCCCGTCACGTCATGAAAGTCGGTAACACCCGAAGCCGGTGGCCTAACCCCCNTGT
GGGGAGGAGCCGT

>J45 / *Georgenia satyanarayanai*

GTCGAACGATGAAGCCCAGCTTGCTGGGTGGATTAGTGGCGAACGGGTGAGTAACACGTGAGTAACCT
GCCCCTGACTTCGGGATAACTACGAGAAATCGTGGCTAATACCGGATATGACACGTGCCTGCATGGGTG
TGTGTGGAAAGATTTATCGGTTGGGGATGGGCTCGCGGCCTATCAGCTTGTTGGTGGGGTGATGGCCTA
CCAAGGCGACGACGGGTAGCCGGCCTGAGAGGGTGACCGGCCACACTGGGACTGAGACACGGCCCAG
ACTCCTACGGGAGGCAGCAGTGGGGAATATTGCACAATGGGCGCAAGCCTGATGCAGCGACGCCGCGT
GAGGGATGACGGCCTTCGGGTTGTAAACCTCTTTCAGTAGGGAAGAAGCTCTTCGGAGTGACGGTACCT
GCAGAAGAAGCGCCGGCTAACTACGTGCCAGCAGCCGCGGTAATACGTAGGGCGCAAGCGTTGTCCGG
AATTATTGGGCGTAAAGAGCTCGTAGGCGGTTTGTCGCGTCTGCTGTGAAAACGCAAGGCTTAACCTTGC
GCCTGCAGTGGGTACGGGCAGACTAGAGTGCGGTAGGGGAGACTGGAATTCCTGGTGTAGCGGTGGAA
TGCGCAGATATCAGGAGGAACACCGATGGCGAAGGCAGGTCTCTGGGCCGTTACTGACGCTGAGGAGC
GAAAGCATGGGGAGCGAACAGGATTAGATACCCTGGTAGTCCATGCCGTAAACGTTGGGCACTAGGTGT
GGGATCCATTCCACGGGTTCCGTGCCGCAGCTAACGCATTAAGTGCCCCGCCTGGGGAGTACGGCCGCA
AGGCTAAAACTCAAAGGAATTGACGGGGGCCCGCACAAGCGGCGGAGCATGCGGATTAATTCGATGCA
ACGCGAAGAACCTTACCAAGGCTTGACATACACCGGAAAAGTGCAGAGATGTGCTCCCCGTAAGGTCGG
TGTACAGGTGGTGCATGGTTGTCGTCAGCTCGTGTCGTGAGATGTTGGGTTAAGTCCCGCAACGAGCGC
AACCCTTGTCCTGTGTTGCCAGCACGTAATGGTGGGGACTCATAGGAGACTGCCGGGGTCAACTCGGAG
GAAGGTGGGGATGACGTCAAATCATCATGCCCCTTATGTCTTGGGCTTCACGCATGCTACAATGGCCGGT
ACAAAGGGCTGCGATACCGCGAGGTGGAGCGAATCCCAAAAAGCCGGTCTCAGTTCGGATTGGGGTCT
GCAACTCGACCCCATGAAGTCGGAGTCGCTAGTAATCGCAGATCAGCAACGCTGCGGTGAATACGTTCTC

GGGCCTTGTACACACCGCCCGTCACGTCATGAAAGTCGGTAACACCCGAAGCCGGTGGCCCAACCCCTTG
TGGGAGGGAGCTG

>J46 / *Bacillus fermenti*

ATACATGCAGTCGAGCGGACTTTTAAAAGCTTGCTTCTCAAAAGTTAGCGGCGGACGGGTGAGTAACAC
GTGGGCAACCTGCCTGTAAGACTGGGATAACTCCGGGAAACCGGGGCTAATACCGGATAATTCATTTCCT
CTCATGAGGGAATGCTGAAAGACGGTTTCGGCTGTCACTTACAGATGGGCCCGCGGCGCATTAGCTAGT
TGGTGAGGTAACGGCTCACCAAGGCCACGATGCGTAGCCGACCTGAGAGGGTGATCGGCCACACTGGG
ACTGAGACACGGCCCAGACTCCTACGGGAGGCAGCAGTAGGGAATCTTCCGCAATGGACGAAAGTCTG
ACGGAGCAACGCCGCGTGAGCGATGAAGGCCTTCGGGTCGTAAAGCTCTGTTGTTAGGGAAGAACAAG
TACCGGAGTAACTGCCGGTACCTTGACGGTACCTAACCAGAAAGCCACGGCTAACTACGTGCCAGCAGC
CGCGGTAATACGTAGGTGGCAAGCGTTGTCCGGAATTATTGGGCGTAAAGCGCGCGCAGGTGGTTCTTT
AAGTCTGATGTGAAAGCCCACGGCTCAACCGTGGAGGGTCATTGGAAACTGGGGAACTTGAGTGCAGA
AGAGGAAAGTGGAATTCCACGTGTAGCGGTGAAATGCGTAGAGATGTGGAGGAACACCAGTGGCGAA
GGCGACTTTCTGGTCTGTAACTGACACTGAGGCGCGAAAGCGTGGGGAGCGAACAGGATTAGATACCCT
GGTAGTCCACGCCGTAAACGATGAGTGCTAAGTGTTAGAGGGTTTCCGCCCTTTAGTGCTGCAGCAAAC
GCATTAAGCACTCCGCCTGGGGAGTACGGCCGCAAGGCTGAAACTCAAAGGAATTGACGGGGGCCCGC
ACAAGCGGTGGAGCATGTGGTTTAATTCGAAGCAACGCGAAGAACCTTACCAGGTCTTGACATCCTCTGA
CACCTCTGGAGACAGAGCGTTCCCCTTCGGGGGACAGAGTGACAGGTGGTGCATGGTTGTCGTCAGCTC
GTGTCGTGAGATGTTGGGTTAAGTCCCGCAACGAGCGCAACCCTTGTCCTTAGTTGCCAGCATTCAGTTG
GGCACTATAAGGAGACTGCCGGTGACAAACCGGAGGAAGGTGGGGATGACGTCAAATCATCATGCCCC
TTATGACCTGGGCTACACACGTGCTACAATGGATAGAACAAAGGGCAGCGAAGCCGCGAGGTGAAGCA
AATCCCATAAATCTATTCTCAGTTCGGATTGCAGGCTGCAACTCGCCTGCATGAAGCCGGAATCGCTAGT
AATCGCGGATCAGCATGCCGCGGTGAATACGTTCCCGGGCCTTGTACACACCGCCCGTCACACCACGAG
AGTTTGTAACACCCGAAGTCGGTGGGGTAACCTTTTAGGGGCCAGCCGCCT

>J50 / *Fictibacillus phosphorivorans*

TCGAGCGNATCGACGAGGAGCTTGCTCCTCTGATTTACGGCGGACGGGTGAGTAACACGTGGGTAATCT
GCCTGTAANACGGGGATAACTCCGGGAAACCGGGGCTAATACCGGATAACAAGAGAAGATGCATATCTT
CTTTTTGAAAGTCGGTTTCGGCTGACACTTACAGATGAGCCCGCGGCGCATTAGCTAGTTGGTGAGGTAA
CGGCTCACCAAGGCGACGATGCGTAGCCGACCTGAGAGGGTGATCGGCCACACTGGGACTGAGACACG
GCCCAGACTCCTACGGGAGGCAGCAGTAGGGAATCTTCGGCAATGGGCGAAAGCCTGACCGAGCAACG
CCGCGTGAGCGATGAAGGCCTTCGGGTCGTAAAGCTCTGTTGTTAGAGAAGAACAAGTACCAGAGTAAC
TGCTCGTACCTTGACGGTACCTAACCAGAAAGCCACGGCTAACTACGTGCCAGCAGCCGCGGTAATACGT
ACGTGGCAAGCGTTATCCGGAATTATTGGGCGTAAAGCGCGCGCAGGCGGTCTCTTAAGTCTGATGTGA
AAGCCCACGGCTCAACCGTGGAGGGTCATTGGAAACTGGGAGACTTGAGTGCAGGANAGAAAAGTGGA
ATTCCACGTGTAGCGGTGAAATGCGTANAGATGTGGAGGAACACCAGTGGCGAACGCGGCTTTTTGGCC
TGTAACTGACGCTGAGGCGCGAAAGCGTGGGGAGCAAACAGGATTAGATACCCTGGTAGTCCACGCCG
TAAACGATGAGTGCTAGGTGTTGGGGGGTTCCACCCTCAGTGCTGAAGTTAACACATTAAGCACTCCGCC
TGGGGAGTACGACCGCAAGGTTGAAACTCAAAGGAATTGACGGGGGCCCGCACAAGCAGTGGAGCATG
TGGTTTAATTCGAAGCAACGCGAAGAACCTTACCAGGTCTTGACATCCTCTGACCACTCTAGAGATAGAG

>J52 / *Salinibacterium xinjiangense*

GAACGATGAAGCTGGAGCTTGCTCTGGTGGATTAGTGGCGAACGGGTGAGTAACACGTGAGTAACCTG
CCCCTGACTCTGGGATAAGCGCTGGAAACGGCGTCTAATACCGGATACGAGCTTCCACCGCATGGTGAG
GAGTTGGAAAGAATTTCGGTCTGGGATGGACTCGCGGCCTATCAGCTTGTTGGTGAGGTAACGGCTCAC
CAAGGCGACGACGGGTAGCCGGCCTGAGAGGGTGACCGGCCACACTGGGACTGAGACACGGCCCAGA
CTCCTACGGGAGGCAGCAGTGGGGAATATTGCACAATGGGCGCAAGCCTGATGCAGCAACGCCGCGTG
AGGGATGACGGCCTTCGGGTTGTAAACCTCTTTTAGTAGGGAAGAAGCGAAAGTGACGGTACCTGCAGA
AAAAGCACCGGCTAACTACGTGCCAGCAGCCGCGGTAATACGTAGGGTGCAAGCGTTATCCGGAATTAT
TGGGCGTAAAGAGCTCGTAGGCGGTTTGTCGCGTCTGCTGTGAAAACTGGAGGCTCAACCTCCAGCCTG
CAGTGGGTACGGGCAGACTAGAGTGCGGTAGGGGAGATTGGAATTCCTGGTGTAGCGGTGGAATGCGC
AGATATCAGGAGGAACACCGATGGCGAAGGCAGATCTCTGGGCCGTAACTGACGCTGAGGAGCGAAAG
CATGGGGAGCGAACAGGATTAGATACCCTGGTAGTCCATGCCGTAAACGTTGGGAACTAGATGTAGGG
ACCATTCCACGGTTTTCTGTGTCGCAGCTAACGCATTAAGTTCCCCGCCTGGGGAGTACGGCCGCAAGGC
TAAAACTCAAAGGAATTGACGGGGGCCCGCACAAGCGGCGGAGCATGCGGATTAATTCGATGCAACGC
GAAGAACCTTACCAAGGCTTGACATATACGAGAACGGGCCAGAAATGGTCAACTCTTTGGACACTCGTA
AACAGGTGGTGCATGGTTGTCGTCAGCTCGTGTCGTGAGATGTTGGGTTAAGTCCCGCAACGAGCGCAA
CCCTCGTCGTATGTTGCCAGCACGTCATGGTGGGAACTCATATGAGACTGCCGGGGTCAACTCGGAGGA
AGGTGGGGATGACGTCAAATCATCATGCCCCTTATGTCTTGGGCTTCACGCATGCTACAATGGCCGGTAC
AAAGGGCTGCAATACCGTAAGGTGGAGCGAATCCCAAAAAGCCGGTCTCAGTTCGGATTGAGGTCTGCA
ACTCGACCTCATGAAGTCGGAGTCGCTAGTAATCGCAGATCAGCAACGCTGCGGTGAATACGTTCCCGG
GCCTTGTACACACCGCCCGTCAAGTCATGAAAGTCGGTAACACCCGAAGCCAGTGGCCCAACCGCAAGG
AGGGAGCTT

> *J53 / Arthrobacter oryzae*

CATGCAGTCGAACGATGATCCCCAGCTTGCTGGGGTGATTAGTGGCGAACGGGTGAGTAACACGTGAGT
AACCTGCCCTTAACTCTGGGATAAGCCTGGGAAACTGGGTCTAATACCGGATATGACTTCTCATCGCATG
GTGGGGGGTGGAAAGCTTTTTGTGGTTTTGGATGGACTCGCGGCCTATCAGCTTGTTGGTGAGGTAATG
GCTTACCAAGGCGACGACGGGTAGCCGGCCTGAGAGGGTGACCGGCCACACTGGGACTGAGACACGGC
CCAGACTCCTACGGGAGGCAGCAGTGGGGAATATTGCACAATGGGCGCAAGCCTGATGCAGCGACGCC
GCGTGAGGGATGACGGCCTTCGGGTTGTAAACCTCTTTCAGTAGGGAAGAAGCGAAAGTGACGGTACCT
GCAGAAGAAGCGCCGGCTAACTACGTGCCAGCAGCCGCGGTAATACGTAGGGCGCAAGCGTTATCCGG
AATTATTGGGCGTAAAGAGCTCGTAGGCGGTTTGTCGCGTCTGCCGTGAAAGTCCGGGGCTCAACTCCG
GATCTGCGGTGGGTACGGGCAGACTAGAGTGATGTAGGGGAGACTGGAATTCCTGGTGTAGCGGTGAA

ATGCGCAGATATCAGGAGGAACACCGATGGCGAAGGCAGGTCTCTGGGCATTAACTGACGCTGAGGAG
CGAAAGCATGGGGAGCGAACAGGATTAGATACCCTGGTAGTCCATGCCGTAAACGTTGGGCACTAGGT
GTGGGGGACATTCCACGTTTTCCGCGCCGTAGCTAACGCATTAAGTGCCCCGCCTGGGGAGTACGGCCG
CAAGGCTAAAACTCAAAGGAATTGACGGGGGCCCGCACAAGCGGCGGAGCATGCGGATTAATTCGATG
CAACGCGAAGAACCTTACCAAGGCTTGACATGGACCGGACCGGGCTGGAAACAGTCCTTCCCCTTTGGG
GCCGGTTCACAGGTGGTGCATGGTTGTCGTCAGCTCGTGTCGTGAGATGTTGGGTTAAGTCCCGCAACG
AGCGCAACCCTCGTTCTATGTTGCCAGCACGTGATGGTGGGGACTCATAGGAGACTGCCGGGGTCAACT
CGGAGGAAGGTGGGGACGACGTCAAATCATCATGCCCCTTATGTCTTGGGCTTCACGCATGCTACAATG
GCCGGTACAAAGGGTTGCGATACTGTGAGGTGGAGCTAATCCCAAAAAGCCGGTCTCAGTTCGGATTGG
GGTCTGCAACTCGACCCCATGAAGTCGGAGTCGCTAGTAATCGCAGATCAGCAACGCTGCGGTGAATAC
GTTCCCGGGCCTTGTACACACCGCCCGTCAAGTCACGAAAGTTGGTAACACCCGAAGCCGGTGGTCCTAA
CCCCTTGTGGGAGGGAGCTGT

>J56 / *Paenisporosarcina indica*

ATACATGCAAGTCGAGCGGAATGATGAAGAAGCTTGCTTCTTCTGATTTTAGCGGCGGACGGGTGAGTA
ACACGTGGGCAACCTACCTTGTAGATTGGGATAACTCCGGGAAACCGGGGCTAATACCGAATAATCCATT
TCGCTTCATGGCGGAATGTTAAAAGGCGGCGCAAGCTGTCACTGCAAGATGGGCCCGCGGCGCATTAGC
TAGTTGGTAGGGTAACGGCCTACCAAGGCGACGATGCGTAGCCGACCTGAGAGGGTGATCGGCCACAC
TGGGACTGAGACACGGCCCAGACTCCTACGGGAGGCAGCAGTAGGGAATCTTCCACAATGGACGAAAG
TCTGATGGAGCAACGCCGCGTGAGTGAAGAAGGTTTTCGGATCGTAAAACTCTGTTGTAAGGGAAGAAC
ACGTACGAGAGTAACTGCTCGTACCTTGACGGTACCTTATTAGAAAGCCACGGCTAACTACGTGCCAGCA
GCCGCGGTAATACGTAGGTGGCAAGCGTTGTCCGGAATTATTGGGCGTAAAGCGCGCGCAGGCGGTCC
TTTAAGTCTGATGTGAAAGCCCACGGCTCAACCGTGGAGGGTCATTGGAAACTGGGGGACTTGAGTGCA
GAAGAGGAAAGCGGAATTCCACGTGTAGCGGTGAAATGCGTAGAGATGTGGAGGAACACCAGTGGCG
AAGGCGGCTTTCTGGTCTGTAACTGACGCTGAGGCGCGAAAGCGTGGGGAGCAAACAGGATTAGATAC
CCTGGTAGTCCACGCCGTAAACGATGAGTGCTAAGTGTTAGGGGGTTTCCGCCCCTTAGTGCTGCAGCTA
ACGCATTAAGCACTCCGCCTGGGGAGTACGGCCGCAAGGCTGAAACTCAAAGGAATTGACGGGGGCCC
GCACAAGCGGTGGAGCATGTGGTTTAATTCGAAGCAACGCGAAGAACCTTACCAGGTCTTGACATCCCA
CTGACCGGTTTAGAGATAAGCCTTTCCCTTCGGGGACAGTGGTGACAGGTGGTGCATGGTTGTCGTCAG
CTCGTGTCGTGAGATGTTGGGTTAAGTCCCGCAACGAGCGCAACCCTTGATCTTAGTTGCCAGCATTCAG
TTGGGCACTCTAAGGTGACTGCCGGTGACAAACCGGAGGAAGGTGGGGATGACGTCAAATCATCATGCC
CCTTATGACCTGGGCTACACACGTGCTACAATGGACGATACAAAGGGTCGCTAACCCGCGAGGGGGAGC
CAATCCCATAAAATCGTTCTCAGTTCGGATTGTAGGCTGCAACTCGCCTACATGAAGCCGGAATCGCTAG
TAATCGCGGATCAGCATGCCGCGGTGAATACGTTCCCGGGCCTTGTACACACCGCCCGTCACACCACGAG
AGTTTGTAACACCCGAAGTCGGTGAGGTAACCTTTTAGGAGCCAGCCGCCGAAGG
